# Periconceptional folic acid supplementation and sex difference in prevention of neural tube defects and their subtypes in China: results from a large prospective cohort study

**DOI:** 10.1186/s12937-018-0421-3

**Published:** 2018-12-12

**Authors:** Jufen Liu, Zhiwen Li, Rongwei Ye, Jianmeng Liu, Aiguo Ren

**Affiliations:** 1 Institute of Reproductive and Child Health / Key Laboratory of Reproductive Health, National Health Commission of the People’s Republic of China, Peking University, Beijing, 100191 People’s Republic of China; 2 Department of Epidemiology and Biostatistics, School of Public Health, Peking University, Beijing, 100191 People’s Republic of China

**Keywords:** Periconceptional folic acid, Sex difference, Neural tube defects (NTDs), Difference in differences (DID) model

## Abstract

**Background:**

Folic acid (FA) supplementation is known to prevent neural tube defects (NTDs). We examined whether this preventive effect differs by the sex of the infant.

**Methods:**

Data were gathered from a large population-based cohort study in China that evaluated the effects of FA supplementation on NTDs. All births at 20 complete gestational weeks, including live births, stillbirths, and pregnancy terminations, and all NTDs, regardless of gestational age, were recorded. In a northern China province, a total of 30,801 singleton live births to women whose use of FA supplements during the first trimester was known at the time were included in the study. The birth prevalence of NTDs was classified by sex, subtype, and maternal FA supplementation. Male to female rate ratios [RR] and their 95% confidence intervals [CI] were calculated.

**Results:**

A total of 106 NTDs cases were recorded. The overall prevalence of NTDs was 2.5‰ among males and 4.4‰ among females; NTDs were less prevalent among males than among females (RR, 0.58; 95% CI, 0.54–0.63). There was a higher prevalence of anencephaly (RR, 0.34; 95% CI, 0.27–0.43) and spina bifida (RR, 0.73; 95% CI, 0.63–0.84) among females. However, FA supplementation led to significantly greater decreases in the rates of anencephaly (4.8‰) and total NTDs (7.6‰) in females than in males (1.6‰ and 2.8‰, respectively).

**Conclusions:**

FA supplementation successfully reduces the prevalence of NTDs in both male and female infants, although we found a significantly greater decrease in anencephaly and total NTDs in females than in males. How the protective effects of FA supplementation affect the sexes differently needs to be studied further.

## Key messages

- Periconceptional FA use reduces NTD risk, but whether the effect differs by infant sex is unknown.
- Daily FA use significantly decreased anencephaly and total NTDs among both males and females in the Chinese population studied.
- These decreases were significantly greater in females than in males.

## Introduction

Many congenital malformations and birth defects, such as defects of the sex organs, urinary tract defects, gastrointestinal tract defects, are sex specific [1–3]. As early as the 1970s, scientists began to notice much higher rates of neural tube defects (NTDs) in females as in males, especially anencephaly [4, 5]. Almost a decade later, researchers suggested that certain environmental factors may contribute to higher rates of anencephalus in females, in particular [6, 7]. In a study that compared the effectiveness of periconceptional vitamin supplementation for the prevention of NTDs recurrence in England and Ireland, beneficial effects were apparent in both countries, and all but one recurrence occurred in male fetuses [8]. A subsequent population study revealed that females tend to have craniorachischisis, spina bifida involving the upper spine, anencephaly, and encephalocoeles more often than males, while males more often have spina bifida affecting the lower spine [9].

Although these findings support the supposition that the less commonly affected sex may be less responsive to environmental therapy, the different rates of NTDs according to sex are difficult to explain because neural tubes close before sex differentiation occurs [8]. While the sequence of maternal events that take place during the development of a fetus are slightly different depending on the sex of the fetus (at least early in pregnancy), the development of male fetuses may be better synchronized on average with these events than female fetuses, from the point of view of NTD [10]. The pattern of variation between the sexes seems to suggest that there are two causes of anencephaly; 1) an environmental cause that predominantly affects female embryos, or 2) an environmental or genetic cause that seems to affect the sexes in roughly equal numbers [6]. However, this difference could also be due to other reasons including sex differences in genetic risk, epigenetic factors, timing of neural tube formation, fetal loss rates, and susceptibility to environmental influences [11, 12].

Periconceptional supplementation with folic acid (FA) has been shown to dramatically reduce the risk for pregnancies complicated with NTDs in different countries including in northern China [13]. Indeed, a recent meta-analysis concluded that fortifying food with FA reduces the overall incidence of NTDs by about 37% globally [14]; however, how this effect may differ by sex is unclear. In addition, some previous studies on NTDs have lacked data on NTD type and/or the sex affected [15, 16]. Still, others did not find significant differences in the effects of FA according to NTD subtype [17] or tended to generalize the apparent protective effects of folate [18, 19]. Contrarily, a study in Mexico found that administration of 5 mg FA each week to be associated with a 50% reduction in the incidence of NTDs, the main effect was on spina bifida, with a higher reduction in female cases [12]. More recent studies in Chile and Argentina showed that folic acid fortification resulted in a greater reduction of anencephaly and cervico-thoracic spina bifida in females compared to males [20]. However, results are not widely generalizable due to the use of a cross-sectional study, as opposed to a population based study. An animal study demonstrated that high maternal FA levels alter gene expression in cerebral hemispheres in a sex-specific manner [21]. In some other studies, FA fortification decreased the rate of spina bifida in both sexes while anencephaly showed no decrease or the change was questionable [22, 23]. Hence, it remains unclear whether or not the effects of FA supplementation significantly differ by NTD subtype and/or the sex of the fetus.

Therefore, the potential sex differences in the preventive effects of FA supplementation on NTD prevalence were examined in northern China, based on a large cohort study in a public health campaign from 1993 to 1996.

## Methods

Starting in 1993, the Chinese Ministry of Health conducted a public health campaign to evaluate the effects of FA on NTDs in 21 counties in southern (Zhejiang and Jiangsu) and northern (Hebei) China. The methods of the original study are well-described elsewhere [13, 24, 25]. There was no FA fortification, nor vitamin supplementation at the time the study conducted in China, because prenatal vitamin use was not considered part of routine antenatal care and women had little access to any vitamin supplements in the project areas at the time [24]. No folate related genotypes or family history of NTD were collected.

A well-organized pregnancy-monitoring system (PMS) was established to collect principal records of prenatal care and demographic information. All women who were preparing for marriage or who became pregnant in the project counties were registered. Women who registered with the PMS between October 1993 and September 1995, and who delivered by 31 December 1996, from five counties in northern China were included. There were 36,144 pregnancies among the enrolled subjects (37425), 97% of all pregnancies [13]. The enrolled women were advised to take a pill containing 400 μg FA every day, starting at the time of registration with the PMS and continuing until completion of the first trimester of pregnancy. If women consented to take FA, the pills were distributed at the time of registration. At the end of each month, health workers recorded the dates of all menstrual periods and how many pills remained in each bottle (if taking pills). A total of 31,960 women with detailed FA supplementation information were included in this analysis.

### Ethics, consent and permissions

The project was approved by the institutional review boards of the US Centers for Disease Control and Prevention and Peking University Health Science Center. All women who took pills provided oral informed consent.

### Definition of FA use

The classification and patterns of FA consumption were the same as previously reported [13]. Women who took FA pills at any time from the registration period until the end of the first trimester of pregnancy were classified as FA users. Women who did not agree to take FA or who were registered during the second trimester of pregnancy (i.e., did not have the opportunity to start taking FA by the end of the first trimester) were considered non-users. Three patterns of FA use were derived based on the start and stop date of supplement use. The groups included periconceptional use (Start: before LMP; Stop: at the end of the first trimester), preconceptional use (Start: before LMP; Stop: before LMP) and postconceptional use (Start: after LMP but within the first trimester; Stop: at the end of the first trimester). Compliance was computed as the percentage of folic acid pills that were taken as compared with the number that could have been taken; ≥80% was regarded as high compliance.

### Identification of NTDs

NTDs were identified through a birth defects surveillance system [13]. This system, which was established in January 1993, collects detailed data on infants and fetuses with external structural birth defects. Live-born infants with birth defects are included in the surveillance system if they have a gestational age of at least 20 weeks and a birth defect diagnosed by 6 weeks of age. Photographs of every infant born with a suspected birth defect and all stillborn infants, whether or not a structural abnormality were collected for diagnosis. Information on all pregnancies, even those with gestations of less than 20 weeks that were electively terminated after the prenatal diagnosis of any birth defect, was also collected. Three pediatricians who were unaware of the women’s pill-taking status independently reviewed the reports and photographs and assigned standard diagnostic codes; a clinical geneticist from USA validated the diagnoses. The NTDs included in our analyses were anencephaly (including iniencephaly, craniorachischisis,), spina bifida, and encephalocele. The prevalence of NTDs by infant sex was also analyzed by NTDs subtype. As anencephaly and spina bifida share causes [6], the prevalence of open NTDs was also determined.

### Statistical analysis

Age, body mass index (BMI), ethnicity, education, occupation, and parity between the groups of women who had and had not taken FA, were compared. The treatment and control groups were compared to determine differences in mean body mass index (BMI), ethnicity, education, occupation, and parity using chi-square analysis and t-tests. We calculated the prevalence of NTDs and subtypes according to the patterns of FA intake. Male to female rate ratios [RR] and their 95% confidence intervals [CI] were calculated. Relative Rate of Prevalence decreased (%) = [(Prevalence of group without FA supplementation -Prevalence of group with FA supplementation)/ Prevalence of group without FA supplementation]*100%.

A difference in differences (DID) model was used to compare differences in the changes in prevalence of NTDs according to maternal FA use, and differences between FA and no-FA groups [26, 20]. All data were analyzed using the SPSS software (v. 20.0; SPSS, Chicago, IL, USA). A two-tailed *p* ≤ 0.05 was considered statistically significant.

## Results

Of the 31,960 women, we excluded 137 (0.4%) women with twins and multiple births, 958 (3.0%) women with unknown parity, and 81 (0.3%) infants with ambiguous or unknown sex. Hence, 30,801(96.4%) women were included in our analyses. Table 1 shows select characteristics of the participants according to periconceptional use of FA by sex of infant. A total of 18,201 (59.0%) women took FA during the periconceptional period and 12,664 (41.0%) had not taken FA at all. Women who took FA were on average 2.3–2.4 years younger and their BMIs were 0.3–0.4 kg/m^2^ lower than those who did not take it. Women who took FA supplements were more likely to be primiparous, factory workers, and to have higher educational levels. There were no significant differences for ethnicity between mothers of male and female infants.

**Table 1:** Characteristics of subjects by periconceptional folic acid use and sex of infant

Characteristics	Male infant					Female infant				
	Women with folic acid (*n* = 9497)		Women without folic acid (*n* = 6644)		*P* value	Women with folic acid (*n* = 8652)		Women without folic acid (*n* = 6008)		*P* value
	n(SD)	%	n(SD)	%		n(SD)	%	n(SD)	%	
Age at pregnancy, years	24.5(2.5)		26.8(3.9)		< 0.001	24.5(2.4)		26.9(4.0)		< 0.001
Body mass index, kg/m^2^	21.2(2.0)		21.5(1.9)		< 0.001	21.2(2.0)		21.6(1.9)		< 0.001
Primiparous	9078	95.6	4211	63.4	< 0.001	8297	95.9	3758	62.5	< 0.001
Han ethnic group	9258	99.0	6544	99.2	0.123	8395	98.9	5914	99.2	0.092
Education										
High school or higher	869	9.3	505	7.7	0.001	773	9.1	442	7.4	0.001
Junior high school	7219	77.4	5212	79.0		6563	77.5	4704	79.0	
Primary school or lower, or unknown	1239	13.3	882	13.4		1127	13.3	811	13.6	
Occupation										
Farmer	8180	86.1	6058	91.2	0.000	7424	85.8	5494	91.4	< 0.001
Factory worker	944	9.9	443	6.7		836	9.7	380	6.3	
Other or unknown	373	3.9	143	2.2		392	4.5	134	2.2	

Values for some characteristics may not be equal to total numbers in each group because of missing values

*SD* standard deviation

A total of 106 NTD cases were recorded: 45 anencephaly, 54 spina bifida, and 7 encephalocele. The overall prevalence of NTDs was 2.5‰ among males and 4.4‰ among females. Anencephaly had a higher prevalence among females (2.2‰) than males (0.7‰), regardless of FA supplementation. NTDs were significantly more prevalent in female infants of mothers who did not take FA supplement (8.8‰) than male infants (4.2‰) of such mothers. The rates were 1.4‰ for males and 1.3‰ for females in mothers who took FA supplement (Table 2).

**Table 2:** Prevalence of NTDs by sex and folic acid supplementation in northern China, 1993–1995 (per 1000 births)

Folic acid	N	Open NTDs			Anencephaly			Spina Bifida			Encephalocele			Total NTDs		
		Male	Female	Total	Male	Female	Total	Male	Female	Total	Male	Female	Total	Male	Female	Total
Yes^*^	18,201	1.2	1.2	1.2	0.1	0.2	0.2	1.1	0.9	1.0	0.2	0.1	0.2	1.4	1.3	1.3
No	12,664	3.8	8.7	6.1	1.7	5.0	3.2	2.1	3.7	2.8	0.5	0.2	0.3	4.2	8.8	6.4
Total	30,801	2.2	4.2	3.2	0.7	2.2	1.4	1.5	2.0	1.8	0.3	0.1	0.2	2.5	4.4	3.4

*NTD* neural tube defect

^*^Compared to group without folic acid supplementation, *P* < 0.001

The comparison of folic acid supplementation characteristics showed no significant difference in average months of folic acid supplementation, timing, and compliance between male and female infants, see Table 3. Most women took 5.5 months of folic acid tablets, 70% of them were periconceptional use, and 80% of them showed high compliance (≥80%).

**Table 3:** Characteristics of folic acid supplementation by sex of infant in northern China

Characteristics	N	Women with male infants	Women with female infants	*P*-value
Folic acid supplementation (Months)	18,201	5.5(2.4)	5.5(2.4)	0.180
Timing				0.369
Periconception	12,779	70.5	70.4	
Preconception	3623	20.1	19.8	
Postconception	1714	9.7	9.4	
Compliance				0.190
< 80%	3310	17.9	18.6	
≥80%	14,839	82.1	81.4	

Total NTDs were less prevalent among males than among females. For every 1 case of total NTDs among females, only 0.58 cases occurred among males (rate ratio [RR] 0.58; 95% confidence interval [CI], 0.54–0.63). For open NTDs, RR was 0.50 (95% CI, 0.5–0.6). There was a higher prevalence of anencephaly (RR, 0.34; CI, 0.27–0.43) and spina bifida (RR, 0.73; CI, 0.63–0.84) among females, but this trend was only significant among infants born to mothers who did not take supplements (Table 4).

**Table 4:** Male:female rate ratio of NTDs by folic acid supplementation in northern China, 1993–1995

Folic acid	N	Open NTDs		Anencephaly		Spina bifida		Encephalocele		Total NTDs	
		RR	95% CI	RR	95% CI	RR	95% CI	RR	95% CI	RR	95% CI
Yes	18,201	1.00	0.69–1.46	0.46	0.02–8.62	1.14	0.73–1.77	1.82	0.10–34.48	1.08	0.77–1.50
No	12,664	0.43	0.39–0.49	0.33	0.26–0.42	0.58	0.46–0.72	2.71	0.20–37.04	0.48	0.43–0.53
Total	30,801	0.53	0.48–0.57	0.34	0.27–0.43	0.73	0.63–0.84	2.27	0.58–8.59	0.58	0.54–0.63

*NTD* neural tube defect, *RR* rate ratio, 95%CI, 95% confidence interval

In our analyses, the decreases in the rates of anencephaly (4.8‰), open NTDs (7.5‰), and total NTDs (7.6‰) were significantly greater in females than in males (1.6‰, 2.6‰, and 2.8‰, respectively) (Table 5).

**Table 5:** Change in prevalence rates of NTDs due to FA supplementation by NTD type and sex in northern China, 1993–1995 (per 1000 births)

Change	Male	Female	Female–Male DID Value	*P* value
	rate	rate		
Open NTDs	2.6	7.5	4.9	0.000
Anencephaly	1.6	4.8	3.2	0.000
Spina bifida	1.1	2.7	1.6	0.083
Encephalocele	0.2	0.1	−0.1	0.587
Total NTDs	2.8	7.6	4.8	0.000

Rate of prevalence decrease = Prevalence of group without FA supplementation – Prevalence of group with FA supplementation

*NTD* neural tube defect, *FA* folic acid

## Discussion

NTDs are serious birth defects that occur during early pregnancy and affect the spine (e.g., spina bifida) and the brain (e.g., anencephaly). A large number of studies have found that 50–70% of these defects can be prevented by women consuming sufficient FA daily before conception and throughout the first trimester of pregnancy [13, 27, 28]. In our study, 41% of women didn’t take folic acid supplementation, among those who took folic acid, the compliance≥80% was 81%, which indicated half of mothers in our study achieved sufficient FA daily intake. Based on a large prospective cohort study, we found that FA supplementation had beneficial effects for the prevention NTDs in both males and females. However, our most important finding is that the effect was much stronger among females, both for open NTDs (including anencephaly and spina bifida) and total NTDs. The prevalence of NTDs in females was 7.6‰ lower compared to a 2.8‰ reduction in males among infants born to mothers who took FA versus mothers who did not. Similarly, the prevalence of anencephaly in females was 4.8‰ lower compared to 1.6‰ reduction in males among infants born to mothers who took FA versus mothers who did not.

Since many countries have adopted mandatory fortification of cereal grain products or dietary supplements containing FA, the prevalence of spina bifida and anencephaly has declined significantly [22]. Based on eight population-based surveillance systems that included prenatal ascertainment of these types of birth defects in the United States (USA), the number of NTD-affected pregnancies in the United States declined from 4000 in 1995–1996 (before the FA mandate) to 3000 in 1999–2000 (after the FA mandate). This decline highlights the partial success of the USA FA fortification program as a public health strategy [29]. However, few studies have investigated sex differences in the NTD-prevention effects of adequate maternal FA intake.

In China, since a massive nationwide FA supplementation program was started in 2009, an increasing number of women have begun to consume adequate amounts of FA. The program provides FA supplements, free of charge, to all women who have a rural registration (as opposed to urban registration) and who plan to become pregnant [30]. Hence, it is more important than ever to further elucidate the effects of FA supplementation on infants in China, in particular, how these effects may differ by sex.

One previous case-control study, conducted in China in 2006, reported a female predominance among anencephaly cases (RR, 0.49; 95% CI, 0.30–0.79) but not among spina bifida (RR, 0.90; 95% CI, 0.55–1.45) or encephalocele (RR, 1.03; 95% CI, 0.40–2.69) cases [31]. In our study, we found that females had a higher prevalence of anencephaly and spina bifida, particularly among infants whose mothers did not take FA supplements (anencephaly: RR, 0.34; CI, 0.27–0.43; spina bifida: RR, 0.73; CI, 0.63–0.84). In addition, the male–female ratio was higher for anencephaly and spina bifida in the group associated with FA supplements than in the non-FA group. The fact that FA prevented more female anencephaly cases may indicate heterogeneity in the causes of NTDs. One animal study revealed that female embryos tend to have more NTDs than males [32]. This could be an epigenetic phenomenon considering females methylate most of the DNA in the large inactive X chromosome after each cell division. Consequently, methylation needs for other cells may not be met [33]. Genetic and environmental factors contribute to increase sex ratios in exencephaly (an early stage of anencephaly) and spina bifida, due to preferential lethality of females [34]. Burren et al. [35], however, reported that folate deficiency resulted in an increased frequency of cranial NTDs in embryos carrying the Sp^2H^, which carries an intragenic deletion in the Pax3 gene. Remarkable differences between sexes were observed in response to FA supplementation, with no treatment effect observed among females. In contrast, our findings suggest that open NTDs (including anencephaly) in female human embryos are responsive to FA. Other possible explanations include folic acids exertion of a protective effects through varying mechanisms that more strongly impact females. For example, epigenetic X-inactivation, among other mechanisms, may proceed through different pathways in males and females [20].

The present study had several strengths. First, it was based on a well-organized population-based monitoring system, that used quality control to ensure data quality [13], including village health workers distributed, counted and recorded the dates of menstrual periods for women enrolled and the pills to reflect FA supplementation, the independent, blind and standard diagnosis and final validation of NTDs diagnosis. The use of a population-based surveillance system that covered all live births, all stillbirths at 20 or more gestational weeks, and pregnancy terminations after the prenatal diagnosis of any birth defect at any gestational age was a second strength of the study. This could lay the groundwork for more precise estimations of NTDs than hospital-based surveillance systems [36], especially considering that the majority of fetuses with NTDs are terminated following prenatal diagnosis before 28 gestational weeks in China [37]. Third, we obtained data for NTDs before the national FA supplementation program, which could best reflect the natural status of the population. Fourth, we compared the folic acid supplementation characteristics between mothers of male infants and female infants, which showed no significant difference. Our study revealed the sex-specific effects of folic acid supplementation in a large prospective study.

However, a couple limitations should also be noted. One limitation was that we did not include the detailed subtype of spina bifida (e.g., severe vs. mild), which may be heterogeneous in cause. A further study may be useful to address this. In addition, we did not collect information on the diets of women, which could have influenced the results. However, the study area was in northern China, and the majority of participants were of Han ethnicity, thus there may be a traditional northern diet pattern in play and would likely not affect the overall trends observed in the study. The third limitation was no folate related genotypes or family history of NTD was collected.

## Conclusions

Overall, FA supplementation successfully reduces the prevalence of NTDs in both male and female infants, although there is a significantly greater decrease in anencephaly and total NTDs in females than in males. How the protective effects of FA supplementation affect the sexes differently needs to be studied further. In addition to genetic and environmental factors in determining sex ratios, future studies are needed to investigate the importance of epigenetic factors, imprinting effects, methylation status, and the folate supplementation effect pathway in altering NTDs risk.

## Abbreviations

BMI: Body mass index
DID: Difference in differences
FA: Folic acid
NTD: Neural tube defects
PMS: Pregnancy-monitoring system
